# The German National Program on Psoriasis Health Care 2005–2015: results and experiences

**DOI:** 10.1007/s00403-016-1637-8

**Published:** 2016-04-05

**Authors:** M. Augustin, L. Eissing, A. Langenbruch, A. Enk, T. Luger, D. Maaßen, U. Mrowietz, K. Reich, M. Reusch, K. Strömer, D. Thaçi, R. von Kiedrowski, M. A. Radtke

**Affiliations:** Institute for Health Services Research in Dermatology and Nursing (IVDP), University Medical Center Hamburg-Eppendorf (UKE), Martinistr. 52, 20246 Hamburg, Germany; Department of Dermatology, Heidelberg University Hospital, Heidelberg, Germany; Department of Dermatology, University Hospital Muenster (UKM), Münster, Germany; Dermatological Practice Maxdorf, Maxdorf, Germany; Department for Dermatology, Venereology and Allergology, University Hospital Kiel, Kiel, Germany; Dermatological Practice Dermatologikum Hamburg, Hamburg, Germany; Dermatological Practice Tibarg, Hamburg, Germany; Dermatological Practice Mönchengladbach, Mönchengladbach, Germany; Department for Dermatology, Allergology and Venereology, University of Lübeck, Lübeck, Germany; Dermatological Practice Selters, Selters, Germany

**Keywords:** Psoriasis, Health Care Program, Quality of health care, Long-term outcomes

## Abstract

In 2005, the first national psoriasis survey in Germany revealed large deficits in health care particularly in patients with moderate to severe disease. The consecutive goal was to improve health care for psoriasis countrywide. For this, a large-scale national program was initiated starting with a comprehensive analysis of structures and processes of care for psoriasis. Patient burden, economic impact and barriers to care were systematically analyzed. In order to optimize routine care, a S3 guideline, a set of outcomes measures and treatment goals, were developed. Implementation was enforced by the German Psoriasis Networks (PsoNet) connecting the most dedicated dermatologists. The annual National Conference on Health Care in Psoriasis established in 2009 consented National Health Care Goals in Psoriasis 2010–2015 and defined a set of quality indicators, which are monitored on a regular basis. Currently 28 regional networks including more than 800 dermatologists are active. Between 2005 and 2014 7 out of 8 quality indicators have markedly improved, and regional disparities were resolved. e.g., mean PASI (Psoriasis Area Severity Index) dropped from 11.4 to 8.1 and DLQI (Dermatology Life Quality Index) from 8.6 to 5.9. A decade of experience indicates that a coordinated nationwide psoriasis program based on goal orientation can contribute to better quality of care and optimized outcomes.

## Introduction

Psoriasis is a chronic, genetically disposed immune disorder, which primarily manifests on the skin [23, 32]. Psoriasis can occur at every age and is accompanied by inflammation, reddening and desquamation of the skin. In Germany, 1-year-prevalence of psoriasis is about 2.5 % in the population [12], and about 0.7 % in children [5], resulting in approximately 2 million people affected by psoriasis, including roughly 350,000 children. About 400,000 patients (25 %) [8] suffer from moderate to severe forms and thus show a particularly high need for intervention. Nail involvement in Germany affects about 40 % [10] and psoriatic arthritis approximately 20 % of patients consulting a dermatologist [45, 47]. The disease is accompanied by an exceptionally high level of strain, caused by physical symptoms such as feelings of tightness, itching and pain, as well as psychosocial burden like stigmatization and chronic disease course [51, 52]. From a societal perspective, there is also a considerable socio-economic burden [53]. Chronic patient burden can result in irreversible cumulative life course impairment [33]. The disease burden and the patient’s life course impairment trigger a high need for health care and require early intervention in patients affected by disease. Thus, treatment should follow patient needs on the one hand [21, 29] and evidence-based guidelines on the other [34, 42].

Since the introduction of the first biological antipsoriatic drugs in the year 2005, health infrastructure and quality of health care have been the focus of intensive research in Germany [16]. More than 30 nationwide projects have been conducted within the last 10 years in order to evaluate, explain and improve health care for psoriasis. The outcomes of these large-scale activities have been evaluated on a regular basis.

The intention of this publication is to summarize the course of the national health care program for psoriasis in Germany between 2005 and 2015 and provide recent outcomes data on the goals achieved.

## Research goals

### The national program on psoriasis care was based on the following questions

1. How is the need and the quality of health care for psoriasis in Germany?
2. How can the quality of health care for psoriasis be improved?
3. Which ways of action are to be used?
4. Which is the long-term-outcome of the national psoriasis health care program assessed by quality indicators?

## Methods

**Fig. 1:**
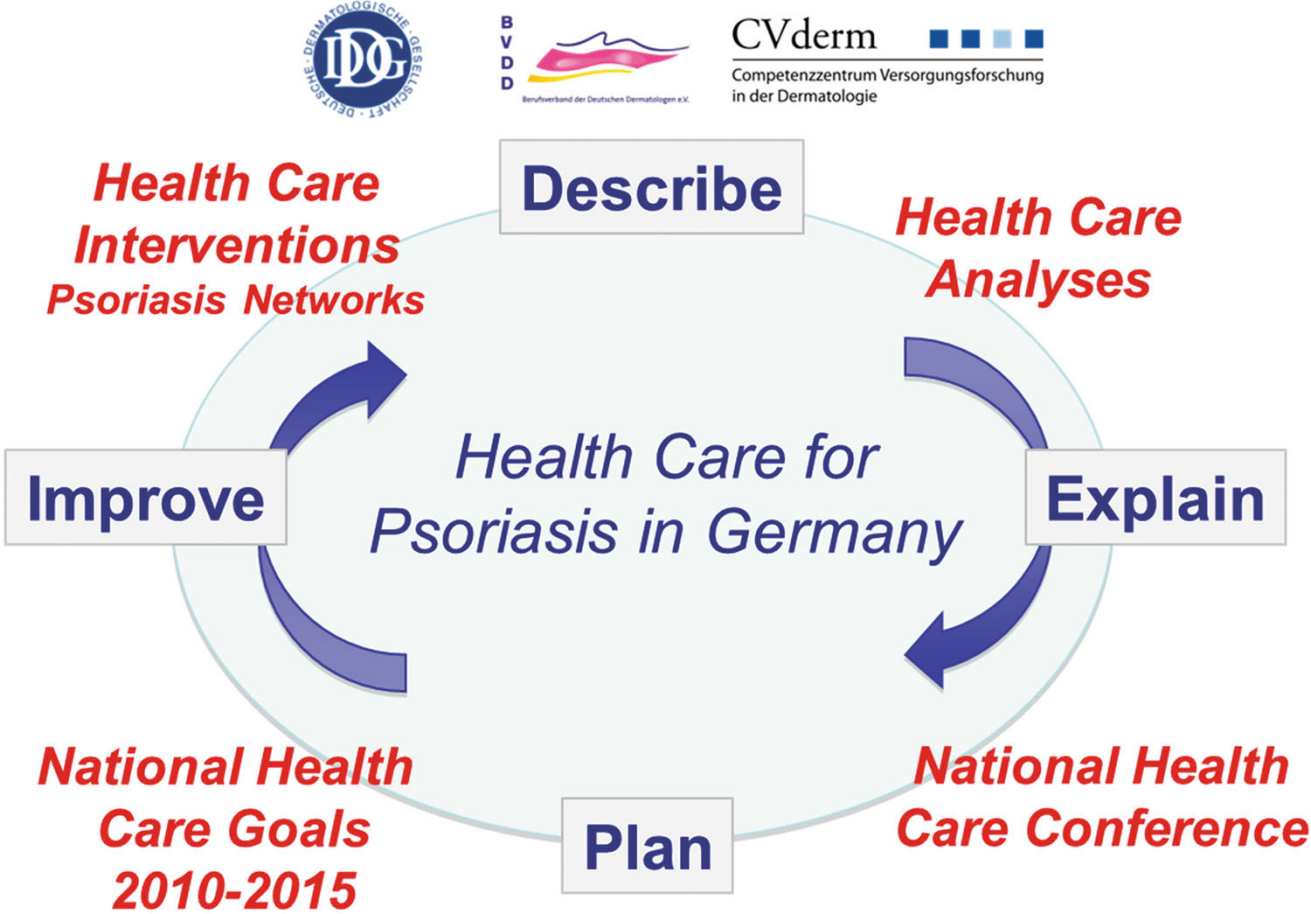
Agenda of the National Program for Psoriasis Health Care 2005–2015

The national psoriasis health care program included the following steps (Fig. 1):

1. Measuring disease burden, needs, quality and outcomes of health care for psoriasis
2. Definition of health care goals
3. Intervention program for improving health care
4. Evaluation program

Overall, the questions raised in the program were addressed in 31 single studies (Table 1). Key results are presented in this paper.

**Table 1:** Series of Health Care Studies in the German National Psoriasis Program, conducted at the German Center for Health Services Research in Dermatology (CVderm) in 2005 - 2015

CVderm 2005–2015		National health care goals addressed				
		1	2	3	4	
Study	Topic	QoL	PsA	Com.	Childr.	HSR
PsoAdhere	Identification and training of adherence in patients with psoriasis	x				
PsoAMNOG	Evaluation of the German AMNOG drug assessment					x
PsoArt	Screening on psoriatic arthritis		x			
PsoBarrier	Exploration of barriers for guideline-compliant health care in psoriasis and development of strategies	x	x	x	x	
PsoBest	The German Psoriasis Registry	x	x	x		
PsoBest-kid	The German Psoriasis Registry, module for children	x		x	x	
PsoCare 1 + 2	SHI health care study on children with psoriasis and atopic eczema	x		x	x	x
PsoCare 3 + 4	SHI health care study on adults with psoriasis and atopic eczema	x		x	x	x
PsoCity	Psoriasis in the population-based Hamburg City Health Study	x	x	x		x
PsoCom	Standards and effectiveness of screening for comorbidity			x		
PsoComp	Patient-relevant endpoints in psoriasis	x				x
PsoCort	Use of corticosteroids in psoriasis: Metaanalysis	x				x
PsoCost	Cost-of-illness and budget impact in psoriasis	x				x
PsoDrop	Analysis of drug survival and persistance in real-world therapy for psoriasis (data from PsoBest)	x	x	x	x	
PsoOdyssee	Development and application of a comprehensive modeling system to predict and quantify processes and outcomes on a national level	x	x		x	x
PsoEpi	Epidemiology of psoriasis in Germany	x				x
PsoEU	Evaluation of the psoriasis health care structures, processes and quality in Europe: Survey in 38 countries	x	x	x	x	x
PsoGoal	Effectiveness of treatment goals in psoriasis	x	x	x	x	x
PsoGuide	Hurdles of guideline-compliant treatment of psoriasis in Germany	x	x	x		x
PsoHarm	Methods for identifying patient harm in systemic treatment	x	x			
PsoHead	Prevalence of scalp psoriasis in Germany	x				x
PsoHealth 1–3	National health care studies for psoriasis 2005, 2007, 2014	x				x
PsoKid	Psoriasis health care in children				x	
PsoLife	Long-term drug survival in psoriasis	x				x
PsoLong	Characteristics of topical long-term treatment for psoriasis in German routine care	x			x	
PsoMetrics	Outcomes, measurement and treatment goals in psoriasis			x		
PsoMod	Modelling of long-term course of psoriasis and psoriatic artrithis	x	x	x		x
PsoNet	German Psoriasis Networks	x			x	x
PsoPharm	National health care studies for psoriasis in pharmacies 2009	x	x	x	x	
PsoPlus	Benefits of membership in a psoriasis patient advocacy group: RCT	x				x
PsoPrefer	Patient preferences in psoriasis therapy	x				x
PsoPRO	Methodology of patient outcomes measurements	x	x	x	x	
PsoRapid	Optimising time to responds in psoriasis treatment	x	x			x
PsoReal	Health-care from the perspective of patient groups in Germany	x				x
PsoSat	Patient satisfaction and treatment optimization	x				
PsoSpecial	Benefits of psoriasis care in a specialized center	x				x
PsoTility	Optimizing use of utility measures in psoriasis	x				x
PsoTop	Topology of psoriasis	x		x		x
PsoVac	Vaccinations in systemic therapies for psoriasis	x	x	x	x	
PsoWeb	Web-based health care studies in psoriasis	x	x	x	x	
PsoWork	Impact of psoriasis on work productivity	x	x			x

Numbers refer to health care goal

*QoL* improving Quality of Life (1)

*PsA* early detection of psoriatic arthritis (2)

*Com.* early detection of comorbidity (3)

*Childr.* improved quality of care for children (4)

*HSR* specific study on health services research (general goal)

For avoiding bias from selection and from social desiredness, different groups of individuals with psoriasis in distinct settings were addressed. e.g., besides studies in dermatology health care, surveys were conducted in the German patient groups [35] via internet [3] and in a network of German pharmacies [30, 31], all using the same set of items.

In order to follow methodological standards, national guidances on the methodology for quality of life assessment [31], for epidemiological research [34] and for registry research [38] were developed.

## Results

### Measuring prevalence, disease burden, quality and outcomes of health care for psoriasis

The series of projects aimed at characterizing the profile of psoriasis health care and the needs for treatment. In order to define the targets of health care research, a consensus group was established 2005, including dermatologists, health economists and health scientists from the German Society of Dermatology (DDG), the Professional Association of German Dermatologists (BVDD) and the German Center for Health Services Research in Dermatology (CVderm). Based on an internal consensus, the following research topics were identified:

- Prevalence of psoriasis
- Patient relevance (patient burden)
- Clinical relevance (health care consumption)
- Economic impact
- Potential for prevention
- Quality of health care
- Guidelines (availability, use, compliance)
- Access to health care
- Benefits of treatments
- Efficiency of health care
- Gaps and under-/overprovision of care
- Barriers of health care

#### Identifying the prevalence of psoriasis in Germany

Epidemiology of psoriasis was the first key research topic for better health care analysis and planning. In order to control for selection bias and limitations of validity, different approaches were chosen, including:

- Analysis of sick fund (claims) data
- Population-based surveys
- Web-based surveys

The resulting prevalence rates were within the same range with a mean prevalence in the sick fund analyses of 2.5 % [50], in the web surveys of 2.4 % [3]. The large-scale whole-body examinations by dermatologists on 90,880 individuals in more than 400 German companies revealed a point prevalence of 2.1 % [7], fitting to the estimated overall one-year-prevalence of 2.5 %.

#### Measuring disease burden

Patient burden from psoriasis in Germany was evaluated in a series of large-scale cross-sectional studies. Significant quality of life (QoL) losses were detected in the first studies in 2005 [9]. e.g., mean DLQI in patients seeking treatment in German dermatology practices was 8.6, mean PASI 12.0. 34.1 % of patients showed DLQI >10 indicating relevant impairment of QoL. Only 33 % of patients with severe disease received systemic treatments. Moreover, there was large dissatisfaction with and a high perceived burden from treatment. This therapeutic burden was identified as an important predictor of QoL impairment [22].

#### Costs of psoriasis

Two cost-of-illness studies revealed that psoriasis is accompanied by a high socio-economic burden [20]. Annual disease costs in Germany were estimated to be 9000 € for severe cases and 4000–7000 € for mild to moderate forms of psoriasis [53]. Causes for high direct costs are expenses for medication and inpatient treatment; indirect costs arise from absence from work and productivity losses. An additional 2400 € are initiated by comorbid diseases. In Germany, compensation for patients with statutory health insurance with mild psoriasis is less than 500 €, with severe psoriasis approximately 7000 €. Other cost determinants are intangible costs caused by loss of QoL and psychological strain. Further data on patient self-medication—invisible in the claims data—were derived from a survey in the pharmacy networks. Substantial patient co-payments of about 800 € per year were identified.

#### Identification of patient needs

**Fig. 2:**
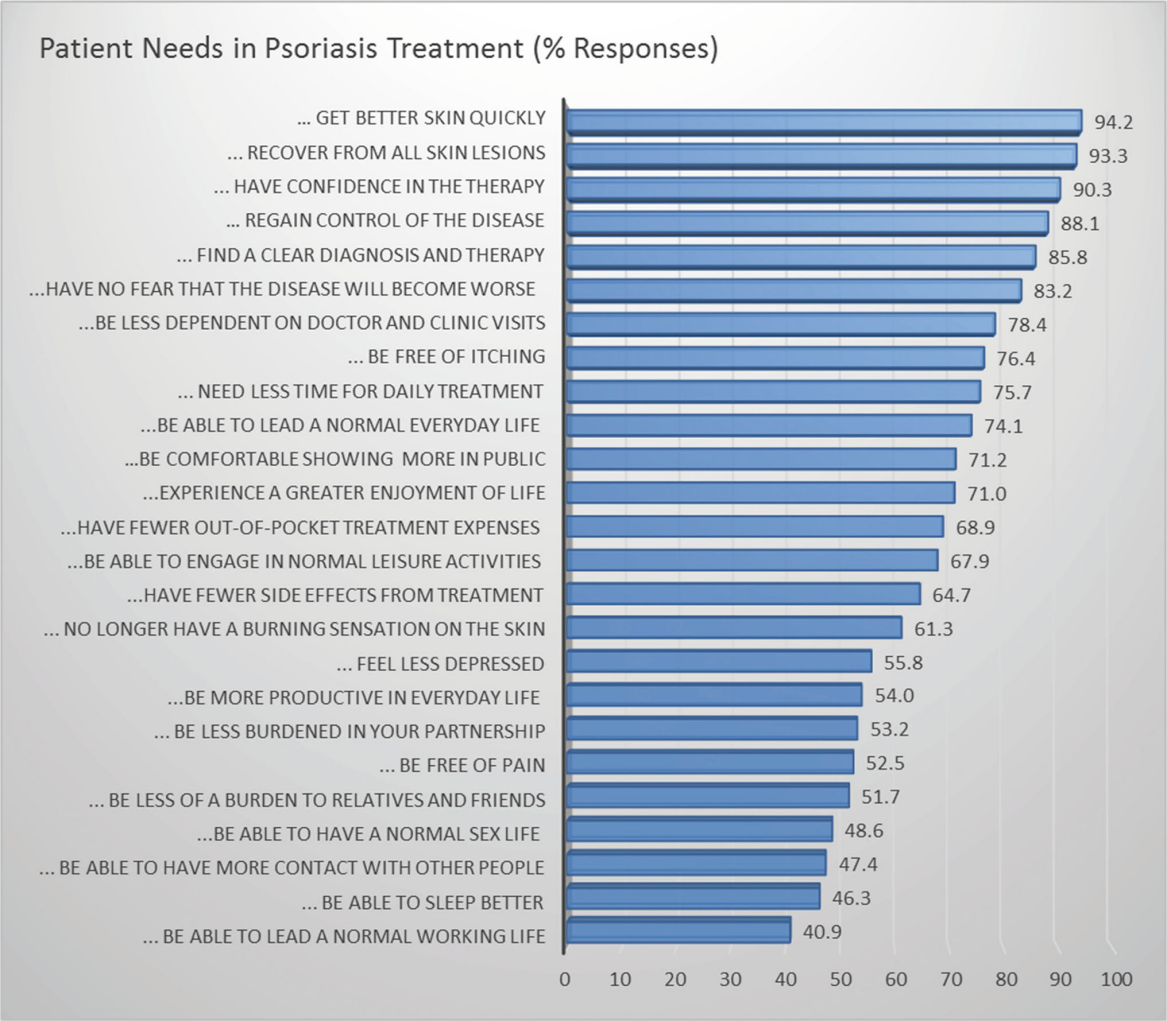
Patient goals and needs from treatment in psoriasis

In order to address more specific support for patients, therapeutic needs were to be identified. Using the Patient Benefit Index (PBI) [15], a broad spectrum of patient-relevant therapeutic needs and potential benefits from treatments were identified (Fig. 2) [21]. Psoriasis patients named 21 out of 25 standardized benefit items to at least 50 %, including clearance of skin lesions, improvement of itching and burning of skin, less time needed for treatment, avoidance of treatment side effects and reduced physician and clinic consultations. Such a broad spectrum of patient needs required specific consideration in the translation of guidelines into clinical practice. In particular, the choice of therapy and the definition of treatment goals should take individual patient preferences into consideration.

#### Definition of health care needs

Due to psoriasis prevalence, disease burden and socio-economic impact, a high need for health care in psoriasis was concluded. Additional demands arise from needs for early detection and treatment of comorbidity: for example, a study in 2005 revealed that more than 80 % of patients with psoriatic arthritis had not yet been adequately diagnosed [47]. Association of psoriasis with obesity, diabetes mellitus, arterial hypertension, dyslipidemia and autoimmune diseases was shown in German population-based epidemiological studies [12], confirming these associations as previously described in other countries. Juvenile psoriasis patients already present with higher prevalence of comorbidity such as diabetes and obesity [9]. For this, a systematic screening and awareness program including the different health care providers was decided as national health care goal.

#### Measuring quality of health care and guideline compliance

- Definition and assessment of quality indicators

Quality measurement depends on standardized criteria. For this, indicators for quality of health care in Germany based on the national S3 guideline were developed in a consensus process [44]. When applied in the first national study PsoHealth1 conducted in 2005, there were striking deficits in most indicators, which markedly improved in the second national study PsoHealth2 2007 [13]. In spite of these improvements, urgent need for further action remained, which resulted in the definition of National Health Care Goals in Psoriasis 2010–2015 [25].

For the evaluation of the national health care quality, nationwide cross-sectional surveys in dermatology practices and clinics were conducted [13]. In each survey, 50 % of the centers derived from previous surveys and 50 % were randomly selected from the list of dermatologists in Germany, including about 3700 practice-based dermatologists and 119 clinical departments. Each center received a set of case report forms for 20 patients, which needed to be included consecutively. For each patient, a patient and a physician questionnaire were filled at the time of presentation in office. Descriptive data analysis was conducted as described previously [13].

(b) Analysis of regional variations

Regional disparities which contradict expected outcomes can be excellent indicators of health care quality. For this, all national health care studies in the German psoriasis program were analyzed by geographical variables. Large disparities in the prescription rates for systemic and biologic drugs were found, although psoriasis population, severity and clinical patterns do not vary significantly [42]. Remarkably, areas with higher prescription rates for systemic drugs also showed better average quality of life and patient benefits from treatment than areas with low use of systemic drugs.

(c) Analysis of treatment patterns in different specialties

Treatment patterns also depended on the specialty consulted. The most striking finding was the widespread use of systemic corticosteroids for psoriasis, especially by internists and general practitioners (GPs), less often by dermatologists [17]. Even when adjusted for comorbidity requiring systemic steroids, these drugs were by far the most frequently used systemic treatment for psoriasis in Germany.

#### Evaluating access to psoriasis health care and treatment variations

Knowledge of the proportion of patients with psoriasis seeking health care in different specialties was crucial for developing targeted health care interventions. Sick fund analyses revealed that in Germany, psoriasis health care is provided to an almost similar extent by dermatologists and general practitioners (GP) (40–50 % each) [6]. Regarding incidental patients, about 65 % start seeking a dermatologist and 38 % a GP. A smaller percentage of adult patients is also treated by other fields of profession, such as rheumatologists in the case of psoriatic arthritis. In pediatric psoriasis, about one-third of children and adolescents with psoriasis are cared for by dermatologists, general practitioners and pediatricians respectively [11]. Large discrepancies with regard to prescribed therapies and quality of health care were observed between the different fields of profession, e.g. pediatricians, unlike dermatologists, rarely prescribed topical Vitamin D analogues for mild psoriasis. And dermatologists, unlike GPs and pediatricians, rarely used systemic steroids. Thus, an obvious need for interprofessional communication on treatment standards was identified.

### Intervention program for improving health care in psoriasis

#### Development and implementation of guidelines

In order to standardize treatment on a national level and to provide maximum quality of care, a national evidence-based S3 guideline on psoriasis treatment for adults was developed and published in 2006 and updated in 2011 [39]. The developing process included patient participation. Concordantly, a patient version of the guideline was published in 2007. Due to the lack of a substantial number of controlled psoriasis studies for children and adolescents, no evidence-based guideline but a consensus paper was developed for the age group below 18 years [54].

#### Patient empowerment

Up-to-date health care includes patient participation in the process of therapeutic decisions (participatory medicine). This concept requires sufficient information and the patient’s understanding of the disease. In order to support patient empowerment, the patient guideline on psoriasis treatment summarizes the content of the professional guideline in appropriate language and is available free of cost. Moreover, a systematic implementation of patient empowerment was started in 2006 in collaboration with the German self-help organization for psoriasis patients (DPB), including annual conjoint meetings between patient groups and dermatologists [14].

#### National Conference on Health Care in Psoriasis and National Health Care Goals in Psoriasis 2010–2015

Steering of health care and harmonization of nation-wide health care goals are consented at the annual “National Conference on Health Care in Psoriasis” in Germany [19]. Delegates of the conference are the executive boards of the dermatological society and the professional association as well as representatives of the regional psoriasis networks. This conference passed the “National Health Care Goals in Psoriasis 2010–2015” in 2009. Specifically, these goals are:

1. Patients with psoriasis have a good quality of life
2. Psoriatic arthritis is diagnosed timely
3. Comorbidity in psoriasis is diagnosed timely
4. Juvenile psoriasis is diagnosed and treated timely

These goals are a self-commitment of dermatologists for the achievement of a measurable, good quality of care as a general goal. All goals were differentiated in sub-goals with a threshold to be achieved by 2015, e.g. the proportion of patients with severe psoriasis receiving systemic drugs could be increased from 33 % in 2005 to 65 % by 2015.

#### Introduction of outcomes tools and treatment goals

One of the most important components of improved and efficient health care is the implementation of tools for the measurement of outcomes. Health care and treatment goals can be set and used as standard only if it is possible to validly measure them. Consequently, training and measurement tools were developed, such as the “PASImeter” to measure the severity degree, the “PsAmeter” for detection and assessment of psoriatic arthritis, and the “Comorbimeter” for early diagnosis of comorbid diseases.

Besides national guideline recommendations for appropriate psoriasis treatment, systematic treatment goals were introduced by German experts [48], aiming at effective and efficient therapy for individual patients. The core concept is that therapeutic measures should only be continued as long as there is prospect for disease improvement. More stringent and efficient care is possible if treatment goals related to specific time periods are set. This concept was later transferred to a European consensus document [37, 48]. It could be shown that the use of treatment goals obviously favors patient satisfaction and improves psoriasis outcomes [46].

#### Development of regional psoriasis networks: PsoNet

For nationwide improvement of health care, 28 regional psoriasis networks have been established since 2008 [1]. Within the initiative German Psoriasis Network (PsoNet), dermatologists with special interest and expertise cooperate efficiently. Core elements are their commitment to qualified care according to the S3 guideline, their willingness to cooperate in an interdisciplinary approach, and the participation in health care research projects.

Consequently, the main PsoNet objective is the implementation of the National Health Care Goals in Psoriasis 2010–2015 [25] and thus quality of health care improvement on the basis of the S3 guideline. Early detection of psoriatic arthritis and other comorbidities by dermatologists are goals that can only be achieved by close cooperation with other disciplines, with dermatologists as important switch setters. The regional psoriasis networks support the course through facilitation of cooperation, with PsoNet standing for improved care through guideline-compliant use of the entire spectrum of treatment options. Health care goals, like the early detection of comorbidity in psoriasis are also addressed by focus campaign, including a consensus on comorbidity screening [43]. Every dermatologist and cooperating physician of other specialty treating psoriasis or psoriatic comorbidities is invited to join the German Psoriasis Network (PsoNet). Besides the annual national meeting, cross-connection between the regional networks and improved awareness is reached by a biannual magazine called PsoNet Magazin [4] edited by the presidents of the German dermatology societies, the German self-help organization for psoriasis patients (DPB) and the chair of PsoNet which provides internal up-to-date information on psoriasis health care issues. Further information is provided on a regular basis by the website, including a search function for dermatologists specialized in psoriasis [24].

#### Improved patient safety: The German Psoriasis Registry PsoBest

In 2008, The German Psoriasis Registry PsoBest has been established as a comprehensive patient registry for monitoring the long-term course of systemic and biologic therapies [18, 49]. Target parameters are drug safety and effectiveness under routine conditions. While short- and intermediate-term efficacy has been demonstrated with a high level of evidence by a vast number of clinical trials and summarized in the S3 treatment guideline, data on effectiveness, safety and optimum modalities in long-term treatment under everyday conditions are lacking. This gap is bridged by PsoBest which includes real-world patients at the start of systemic or biologic treatment and monitors them irrespective of the treatment course for the subsequent 5 years. During this follow-up, data are collected with standardized physician- and patient questionnaires altogether 12 times in practices as well as 9 times in the interim by mail. Scientific quality is ensured by methods following international guidance; furthermore, the project is supervised by an interdisciplinary scientific advisory board in agreement with the dermatologic and expert associations. PsoBest is part of the European network of psoriasis registries [40]. At present, 691 dermatology practices and 64 hospital outpatient clinics actively participate in PsoBest, reporting almost 4500 patients up to now with about 270 centers providing 90 % of data.

#### Identification of barriers

**Fig. 3:**
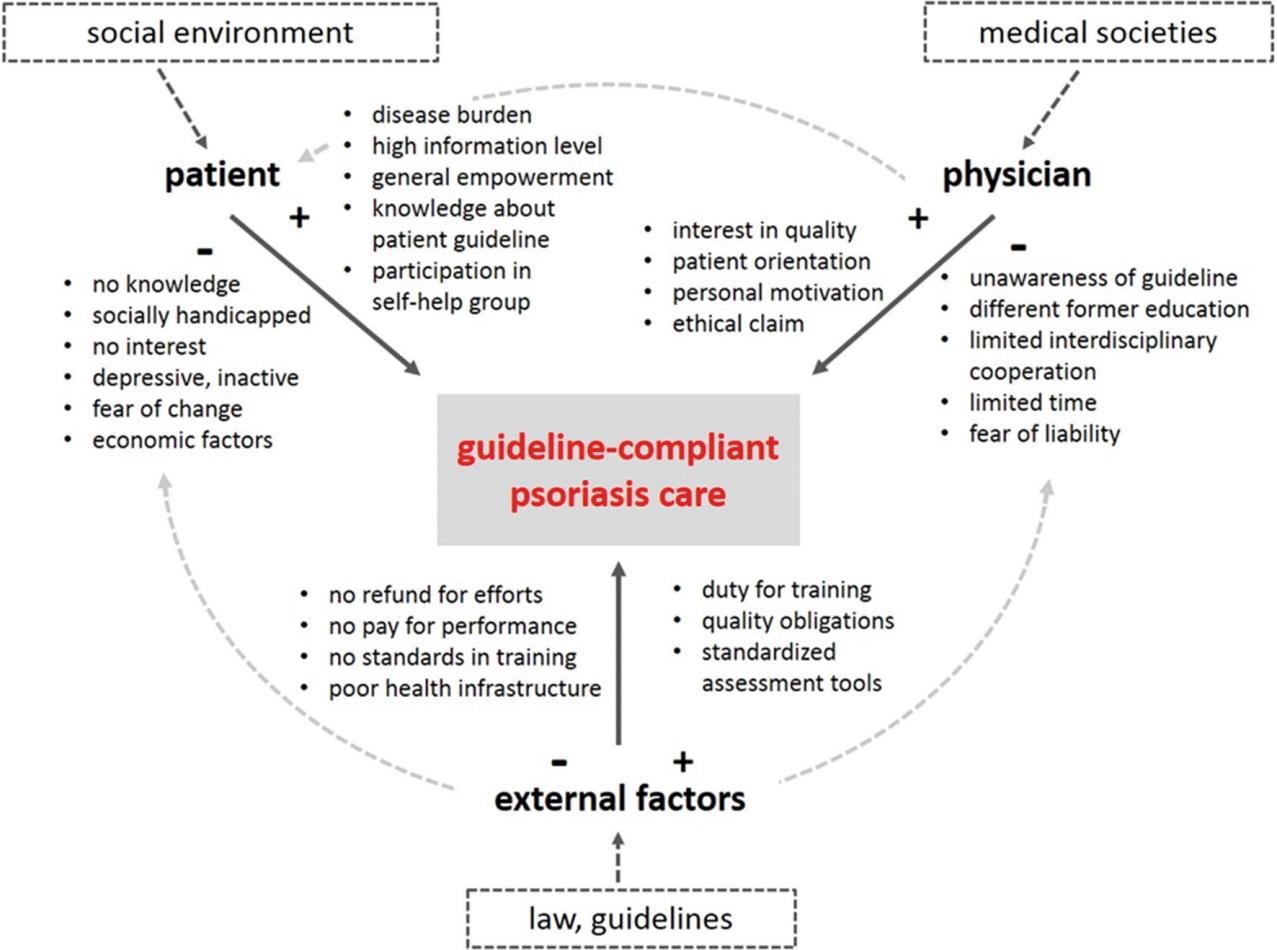
Barriers to guideline-compliant psoriasis care

Restoring quality of life of patients, reducing psychosocial burden and morbidity risks, and at the same time achieving sufficient health care efficiency are the consented goals for psoriasis treatment in Germany. Realization of these goals is led by current research findings that are summarized in the evidence-based S3 psoriasis treatment guideline. One of the health care research objectives is to identify barriers in guideline-compliant care and consequently to contribute to their resolution [27]. Research is oriented along the three-part barrier model with the components “external factors”, “physician”, and “patient” (Fig. 3). All factors have facilitating as well as obstructing effects on barriers and consequently, each of them contribute to quality of health care.

#### Awareness program: World Psoriasis Day

In order to increase awareness for psoriasis as a widespread disease and to support patient needs, the World Psoriasis Day is held worldwide yearly on October 29th since 2005. This day intends to emphasize the issues of 150 million people affected by psoriasis worldwide. In Germany, most regional psoriasis networks organize public events and are available for media on this day which is widely announced on a specific website [26].

#### Certification on quality in psoriasis care

In 2014, the German Society of Dermatologists (DDG) and the Professional Association of German Dermatologists (BVDD) decided to develop a certificate, which outlines dermatologists to have a particularly high expertise in psoriasis care. Quality is controlled by participation in the PsoBest registry. Specific contracts and honoraria by the payers are connected with this certificate.

#### Dissemination of health care data

For optimum improvement of quality of care, continuous information of the health care decision makers and the payers is crucial. In order to disseminate respective facts, the book “Versorgung der Psoriasis in Deutschland: Fakten 2014” (German Health Care on Psoriasis: Facts 2014) was developed, summarizing all information on health care for psoriasis. Moreover, a multi-author special issue of the most common German health political journal "Gesellschaftspolitische Kommentare–gpk" (Societal Political Comments) for the stakeholders in health politics was issued, addressing to more than 6000 decision makers in the national and regional parliaments, health care administrations, sick funds and further bodies.

### Evaluation program for improvement of health care in psoriasis

In order to verify the psoriasis health care program in Germany, the central outcomes and quality indicators of psoriasis care are recorded on a regular basis.

The last evaluation in 2013/2014 has revealed a substantial increase in quality of health care compared to 2005 and 2007. 7 out of 8 indicators significantly improved, and 2 reached the level of the health care goals 2010–2015 prematurely (Table 2) [36], e.g. mean PASI dropped from 11.4 (2005) to 10.1 (2007) and 8.1 (2013), mean DLQI decreased from 8.6 (2005) to 7.5 (2007) and 5.9 (2013). The proportion of patients with PASI >20 declined from 17.8 (2005) to 11.6 (2007) and 9.2 (2013), the proportion of patients with DLQI >10 from 34.0 (2005) to 28.2 (2007) and 21.3 (2013). In the same period the proportion of patients, who had received systematic treatment for psoriasis in the previous 5 years increased from 32.9 % (2005) to 47.4 % (2007) and 59.5 % (2013) and reached the national health care goal. Finally, there was also a decrease in lost work days from 4.9 (2005) to 3.5 (2013).

**Table 2:** Development of the quality of health care for psoriasis in Germany as measured by guideline-derived quality indicators [36]

Indicator	PsoHealth1 (2005)	PsoHealth2 (2007)	PsoHealth3 (2013/14)	Goal by end of 2015 [56]	Trend 2005–2014
*n*	1511	2009	1258		
Mean PASI	11.4	10.1	8.1	<8.0	+^a^
Mean DLQI	8.6	7.5	5.9	<6.0	++^b^
% PASI >20	17.8 %	11.6 %	9.2 %	<10 %	++
% DLQI >10	34.0 %	28.2 %	21.3 %	<15 %	+
% With previous systemic treatment	32.9 %	47.3 %	59.5 %	>65 %	+
% Hospital treatment (past 5 years)	26.9 %	20.1 %	20.1 %	<15 %	(+)^c^
Mean number of days absent from work	4.9	4.0	3.5	<3.0	+

^a^+ = Major improvement

^b^++ = Goal 2015 reached prematurely

^c^(+) = Minor improvement

## Discussion

Psoriasis is a common chronic disease affecting all age groups and leading to substantial patient burden [5, 8, 41]. There is high need for health care, especially in patients with moderate to severe disease or significant comorbidity. In spite of this, in Germany like in many other countries there was a low level of awareness and a marked lack of health care provision when first systematic data were researched in 2005.

In order to better characterize health care for psoriasis and to identify the specific needs for treatment in Germany, a series of health care studies was conducted between 2005 and 2008, which showed the gaps and potential goals of action [55]. The results of these first investigations triggered a systematic national program for improving health care in psoriasis, which included strong collaboration between dermatologists and patients. Major elements were the development of a S3 treatment guideline in 2006, the implementation of regional psoriasis networks by dermatologists in 2008, the establishment of an annual national psoriasis conference in 2009 and the definition of National Health Care Goals in Psoriasis 2010–2015. At the patient level, the initiatives for measuring disease, defining treatment goals and turning to a more patient-centered treatment approach were crucial. By cross-sectional health care studies, the goal achievement was verified and specific measures both on regional and national levels taken. To our knowledge, this is the first such attempt in the field of psoriasis.

The results of the recent health care study PsoHealth3 show that within the period between 2005 and 2014, large nationwide improvements have been achieved with respect to reducing disease severity and burden, improving quality of life and decreasing indirect costs due to reduced work productivity. Regional comparisons were very supportive in identifying the need for action. Nevertheless, gaps in health care provided by dermatologists remain and there are still greater deficits in health care for psoriasis by GPs, pediatricians, and internists. Thus, there is a need for redefining health care goals for the period 2016–2020. Regarding patient safety in systemic therapy, the pharmacovigilance data from The German Psoriasis Registry PsoBest indicate a high level of drug safety without any unexpected safety signals to date [41].

On an international level, a higher level of awareness on the need of better psoriasis health care has emerged as well. For example, a European psoriasis petition was released in 2012 followed by the European White Paper published by dermatology experts and patients with the demand for improved quality of care [2, 28]. Such activities, in particular by patient advocacy groups, have supported the resolution by the World Health Assembly (WHA) from May 2014 [55]. With this initiative, the World Health Organization (WHO) has confirmed the need for action both on the level of awareness, fight against stigmatization and better access to treatments in its member states. This WHA decision has further encouraged patients, dermatologists and other health care professionals to claim better treatment for patients in need.

A limitation of the national psoriasis health care program is its main focus on improving quality of care by dermatologists but not by other specialties. Although they provide the largest proportion of health care for psoriasis in the country [6], it has appeared that further quality improvement programs need to be extended to other caring groups, in particular GPs. A limitation of the evaluation program is that there has not been a randomized controlled study design for the evaluation of specific interventions and the nationwide long-term health care outcomes. However, nationwide health care interventions can hardly be subject of a study design like in single clinical trials. Furthermore, the effects on PASI and DLQI might results from a different selection of patients. In order to minimize this potential confounder, a large number of randomly chosen centers were recruited and consecutive patient inclusion was mandatory.

Regardless of these potential limitations, such a nationwide health care program based evidence-based guidelines, structured care and goal orientation may be an impulse for other health care settings and could be beneficial also in other indications. A first transfer using experience with the psoriasis program was initiated in 2015 with the establishment of a national health care program on skin cancer in Germany [21].
